# Carbon-fiber reinforced PEEK instrumentation for spondylodiscitis: a single center experience on safety and efficacy

**DOI:** 10.1038/s41598-021-81960-8

**Published:** 2021-01-28

**Authors:** Ann-Kathrin Joerger, Ehab Shiban, Sandro M. Krieg, Bernhard Meyer

**Affiliations:** 1 Department of Neurosurgery, Klinikum Rechts der Isar, Technische Universität München, Ismaningerstr. 22, 81675 Munich, Germany; 2 Department of Neurosurgery, Universitätsklinikum Augsburg, Stenglinstr. 2, 86156 Augsburg, Germany

**Keywords:** Neurology, Infectious diseases

## Abstract

Radiolucent carbon-fiber-reinforced (CFR) polyethyl-ether-ether-ketone (PEEK) has been established in spinal instrumentation for oncological reasons. Laboratory data reported comparable bacterial adhesion as titanium. Thus, using of CFR-PEEK spinal instrumentation for spondylodiscitis bases on artifact-free imaging to evaluate therapeutic success. Studies comparing the rate of pedicle screw loosening and relapse of spondylodiscitis following titanium versus CFR-PEEK instrumentation do not exist so far. This study evaluates the rate of pedicle screw loosening and recurrence of spondylodiscitis after CFR-PEEK instrumentation for spondylodiscitis compared to titanium. We conducted a prospective single center study between June 2018 and March 2019 on consecutive 23 patients with thoracolumbar spondylodiscitis. Imaging data was evaluated for screw loosening at a minimum of three months after surgery. A matched-pair analysis was performed using spondylodiscitis cases between 2014 and 2016 using titanium instrumentation for equal localization, surgery, and microorganism class. Among 17 cases with follow-up imaging, six cases (35%) showed screw loosening while only 14% (two patients) with titanium instrumentation were loosened (*p* = 0.004). In both groups the most frequent bacterium was *Staphylococcus aureus*, followed by *Staphylococcus epidermidis*. From the *S. aureus* cases, one infection in both groups was caused by methicillin resistant species (MRSA). No difference was found in the rate of 360° fusion in either group due to matching criteria. As opposed to other indications CFR-PEEK screws show more loosening than titanium in this series with two potentially underlying reasons: a probably stronger bacterial adhesion on CFR-PEEK in vivo as shown by a statistical trend in vitro and instrumentation of spondylytic vertebrae. Until these factors are validated, we advise caution when implanting CFR-PEEK screws in infectious cases.

## Introduction

Carbon-fiber-reinforced (CFR) polyethyl-ether-ether-ketone (PEEK) material has been used successfully for many years in the orthopedic field^1^ and for spinal fusion techniques^2–4^. Results from cadaver and clinical studies exist which show that CFR-PEEK rods can withstand equal stiffness and resistance to motion compared to titanium rods for lumbar fusion^5,6^. For the use of CFR-PEEK instrumentation in primary spinal tumors a low intraoperative complication rate and low rate of pedicle screw loosening has been shown^7^. The advantage of CFR-PEEK material is its radiolucency which reduces artifacts on computed tomography (CT) and magnetic resonance imaging (MRI)^8^, thus making this material more amenable to follow-up analysis. Moreover, CFR-PEEK screws show less radiation interference than titanium screws^9^, so radiotherapy can be applied more precisely. Therefore, CFR-PEEK seems as a promising option for the instrumentation of spinal tumors and metastases, as artifact-free postoperative imaging is essential for radiotherapy planning, for precise application of radiotherapy and for the evaluation of tumor progression.

Artifact-free postoperative images are not only of interest in the oncological field, but also in the treatment of spinal infection. Treatment of spondylodiscitis consists of spine immobilization and antibiotic therapy^10^. To treat patients with spondylodiscitis without intraspinal empyema or neurological deficit conservatively or surgically is still an ongoing international debate^11^. The advantage of surgical treatment using spinal instrumentation is an earlier mobilization of patients and lower complication rates in modern series^12^. Evaluation of therapeutic success is important before termination of the antibiotic therapy. Successful treatment of spondylodiscitis is evaluated by clinical findings such as infection parameters, pain reduction, and by follow-up imaging^13^.

The idea to use CFR-PEEK instrumentation for spondylodiscitis cases origins from the ability to gain artifact-free follow-up imaging, enhancing the evaluation of therapeutic success. Laboratory data showed that CFR-PEEK material harbors statistically no unfavorable bacterial adhesion as titanium does^14^.

However, the use of CFR-PEEK instrumentation in the treatment of spondylodiscitis has not been established yet. Because of the existing laboratory and clinical results for oncological indications, we hypothesized that CFR-PEEK instrumentation is equal or even superior to titanium instrumentation for spondylodiscitis due to artifact-free follow-up imaging.

This study therefore evaluates the first consecutive series of spondylodiscitis patients who underwent CFR-PEEK instrumentation regarding pedicle screw loosening and relapse of spondylodiscitis.

## Methods

### Study design

We conducted a prospective single-center study between June 2018 and March 2019. Patients with thoracic and lumbar spondylodiscitis treated with CFR-PEEK instrumentation were included. Follow-up X-rays, CT- and MRI-scans were evaluated with regard to screw loosening and relapse of spondylodiscitis by a board of seven neurosurgeons. Screw loosening was defined as the manifestation of a halo sign around at least one pedicle screw in the follow-up imaging. A matched-pair analysis was conducted using retrospective data of spondylodiscitis cases between January 2014 and December 2016 using titanium instrumentation. Main criteria for matching were (with decreasing importance) (1) localization: lumbar versus thoracic spine, (2) posterior fusion versus 360° fusion, (3) spinal levels, (4) type of germs detected intraoperatively or by biopsy.

In both groups pedicle screw placement was performed in the same way by 3D-fluoroscopy or CT based navigation.

This study was designed to evaluate safety and efficacy of CFR-PEEK instrumentation for spondylodiscitis. It was conducted according to STROBE guidelines.

### Ethical approval

The study was approved by the ethical committee of our university (“Ethikkommission der Technischen Universität München”, chair: Prof. Dr. G. Schmidt, reference number 229/18s) and conducted in accordance to the Declaration of Helsinki. Written informed consent of all patients was received.

### Statistical methods

To analyze demographic data, the number of 360° fusion/posterior fusion and the reasons for revision surgery Fisher’s exact test was used. To compare patients’ median age student’s-t-test was applied. The number of cases with screw loosening was analyzed using the Chi-square test. *p* < 0.05 was regarded as significant. Statistical analysis was performed by GraphPad Prism 5.0 (La Jolla, California).

## Results

### Patient characteristics

Between June 2018 and March 2019, 24 patients were included in the CFR-PEEK group. One patient was excluded from the study seeing as no bacteria was detected and histopathology definitively excluded an infection. In total, 23 cases with CFR-PEEK instrumentation for spondylodiscitis were analyzed. They were matched with 23 cases with titanium instrumentation. In 16 patients the lumbar spine was affected by spondylodiscitis, in seven patients the thoracic spine. Of the patients with lumbar spondylodiscitis in twelve cases the infection was monosegmental, while in four cases two or more segments were affected. Of the patients with thoracic spondylodiscitis, in five cases it was monosegmental, while in two cases two or more segments were involved. As this was a matching criterion there was no difference between the CFR-PEEK and the titanium group regarding the localization and number of affected segments. Concerning demographic parameters, no statistically significant difference could be detected (Table 1).

**Table 1:** Demographic data of CFR-PEEK and matched titanium group.

	CFR-PEEK group	Matched titanium group	*p* value
**Sex**			
Male (%)	17 (74%)	18 (78%)	1.000
Female (%)	6 (26%)	5 (22%)	1.000
**Age (years)**			
Median (min.–max.)	66 (35–81)	70 (36–88)	0.393
**Comorbidities**			
Alcohol abuse	4	1	0.346
Atrial fibrillation	7	6	1.000
Cancer	12	10	0.768
Cardiac insufficiency	2	3	1.000
Chronic infectious disease	6	2	0.243
Cirrhosis	2	1	1.000
COPD	2	3	1.000
Coronary artery disease	4	5	1.000
Cortison medication	3	1	0.608
Diabetes mellitus	9	6	0.530
i.v. drug abuse	4	0	0.109
Epilepsy	2	1	1.000
Hypertension	13	19	0.108
Hypothyroidism	4	2	0.665
Immunosuppression	0	3	0.234
Nicotine abuse	6	1	0.096
Osteoporosis	0	3	0.233
PAD	3	2	1.000
Parkinson’s disease	0	2	0.489
Pulmonary hypertension	1	0	1.000
Renal insufficiency	8	10	0.763
Rheumatic disease	1	2	1.000
Stroke	3	5	0.700
Valvular heart disease	5	3	0.700

Sex, age and comorbidities of patients of the CFR-PEEK group and the matched titanium group. COPD = Chronic Obstructive Pulmonary Disease, PAD = Peripheral Artery Disease. Sex and comorbidities were compared by Fisher’s exact test. Age by student’s t-test.

### Microbiological spectrum

**Figure 1:**
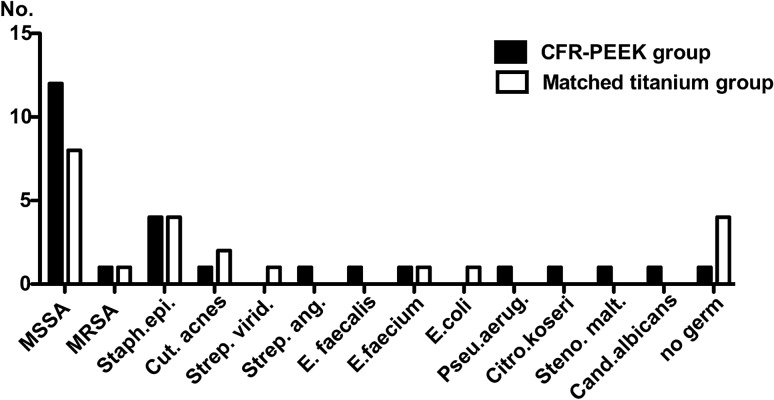
Bacterial spectrum of CFR-PEEK group and matched titanium group. Spectrum of germs causing spondylodiscitis in the CFR-PEEK group and matched titanium group. y-axis designating the number how often a germ was detected. Cand. albicans = *Candida albicans*; Citro. koseri = *Citrobacter koseri*; Cut. acnes = *Cutibacterium acnes*; E. coli = *Eschericha coli*; E. faecalis = *Enterococcus faecalis*; E. faecium = *Enterococcus faecium*; MRSA = methicillin resistant *Staphylococcus aureus*; MSSA = methicillin sensible *Staphylococcus aureus*; Staph. epi. = *Staphylococcus epidermidis*; Steno. malt. = S*tenotrophomonas maltophilia*; Strep. ang. = *Streptococcus anginosus*; Strep. virid = *Streptococcus viridans*; Pseu. aerug. = *Pseudomonas aeruginosa*; No. = number.

The most frequently detected bacterium in both groups was *Staphylococcus aureus* of which one case in both groups was a methicillin resistant species (MRSA), followed by *Staphylococcus epidermidis* in both groups (Fig. 1).

All patients were treated by two weeks of i.v.-antibiotics, followed by ten weeks of oral antibiotics based on the antibiogram of the detected bacterium.

### Surgery

Number of instrumented levels depended on the degree of bony destruction. If this was moderate pedicle screws were placed in the vertebra above and below the affected disc (seven cases with lumbar spondylodiscitis). If there was a significant destruction at least two levels above and below were instrumented (nine cases with lumbar spondylodiscitis, in all cases with thoracic spondylodiscitis). In cases with bechterew’s disease three levels above and below were instrumented.

There was no statistically significant difference in the number of 360° fusion and posterior fusion in both groups (*p* = 0.758) as it was a matching criterion. In the CFR-PEEK group in seven cases vertebral body replacement was performed, in six cases XLIF (extreme lateral intercorporal fusion) and in two cases ALIF (anterior intercorporal fusion). In the eight cases of merely posterior fusion in six cases discectomy and intercorporal fusion with an antibiotic medium and autologous bone was performed. In the titanium group ten cases received intervertebral body replacement and three cases ALIF. In the ten cases with just posterior fusion in eight cases discectomy and intercorporal fusion with an antibiotic medium and autologous bone was performed. 360° fusion, however, was used more frequently in both groups compared to all cases with spondylodiscitis and titanium instrumentation from January 2014 till December 2016. However, this difference, was not statistically significant (*p* = 0.067) (Table 2).

**Table 2:** Number of posterior fusion and 360° instrumentation of CFR-PEEK group, matched titanium group and whole titanium cohort.

	CFR-PEEK group	Matched titanium group	Whole titanium cohort
Posterior instrumentation (%)	7 (30%)	9 (39%)	57 (53%)
360° fusion (%)	16 (70%)	14 (61%)	51 (47%)

Operative strategy used. To compare the number of posterior fusion versus 360° fusion between the CFR-PEEK group and the matched titanium group and between the CFR-PEEK group and the whole titanium group Fisher’s exact test was used. There was no significant difference. CFR-PEEK group versus matched titanium group: *p* = 0.758. CFR-PEEK group versus whole titanium group: *p* = 0.067.

In the CFR-PEEK group in four cases an intraoperative revision of pedicle screws was necessary because of malpositioning, in two cases an accidental durotomy was reported and in one case a pedicle screw broke with the tip left in the pedicle. In the titanium group in six patients intraoperative correction of pedicle screws was necessary and two accidental durotomies occurred.

### Screw loosening

**Figure 2:**
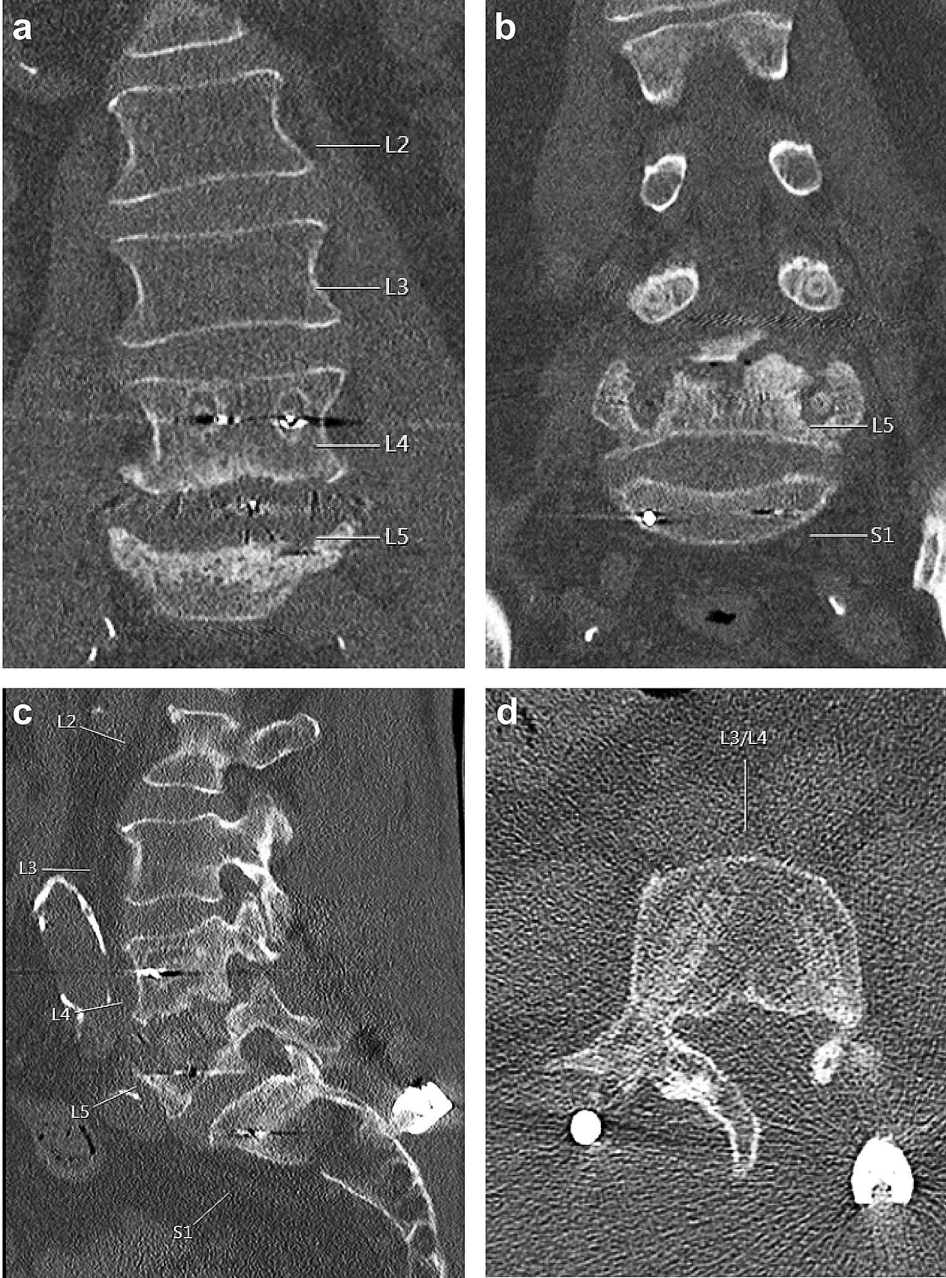
Example 1 of CT-imaging of screw loosening after CFR-PEEK instrumentation. CT-scan of a case of screw loosening of the CFR-PEEK group 128 days post-surgery. CFR-PEEK instrumentation was performed on L4–5–S1–S2 and involved the affected vertebrae on L4/5. L4 and L5 pedicle screws show a halo as a sign for loosening. (**a**,**b**) coronal plane; (**c**) sagittal plane, (**d**) axial plane of L4.

**Figure 3:**
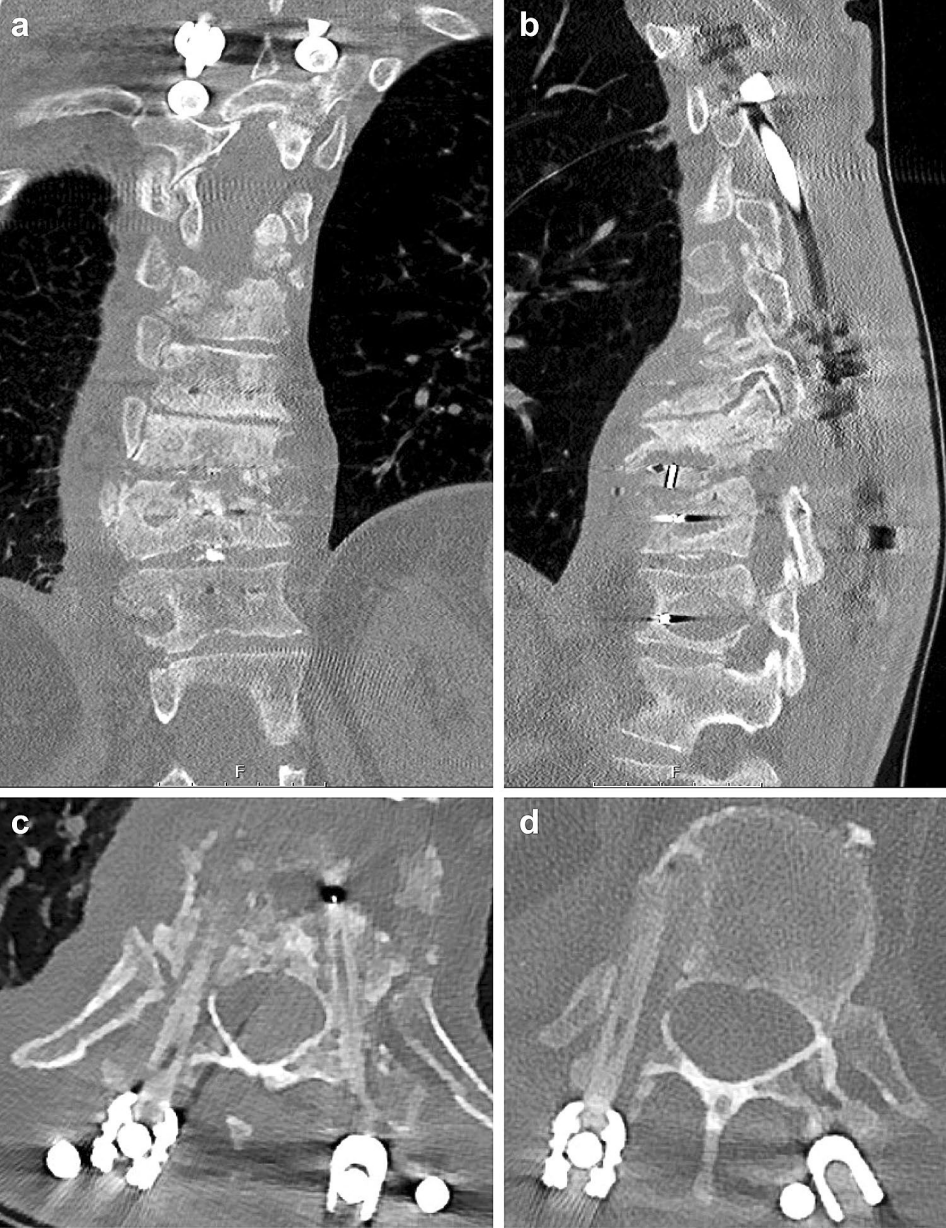
Example 2 of CT-imaging of screw loosening after CFR-PEEK instrumentation. CT-scan of a case of screw loosening after CFR-PEEK instrumentation T5–6–9–10–11–12 involving the affected vertebra on level T11. There a halo signs around the screws of T11 and 12 on the right side as a sign for loosening. (**a**) coronal plane; (**b**) sagittal plane, (**c**,**d**) axial plane of T11 and T12.

In the CFR-PEEK and titanium group, six and nine patients were lost to follow-up, respectively (Table 3). In the CFR-PEEK group screw loosening occurred significantly more frequently than in the titanium group (35% versus 14%, *p* = 0.004) (Table 3, Figs. 2, 3). Mean time of diagnosis of screw loosening was 110 days (± 81) for the CFR-PEEK and 71 days (± 11) for the titanium group. Microbiological spectrum of patients with screw loosening did not differ from the spectrum of the total cohort of patients. As well for CFR-PEEK as for titanium material *S. aureus* was the most frequent bacterium detected within spondylodiscitis cases with loosened screws. *S. epidermidis* was the second most common (Table 4). In the CFR-PEEK group in one case *Cutibacterium acnes* was found in sonication of the explanted material. Initially spondylodiscitis of this case was caused by MSSA (Table 5). In the titanium group no bacterium was detected in sonication of the explanted material (Table 6).

**Table 3:** Rate of screw loosening of CFR-PEEK group and matched titanium group.

	CFR-PEEK group	Matched titanium group
Screw loosening	6 (35%)	2 (14%)
No screw loosening	11 (65%)	12 (86%)
Unknown	6	9

Number of cases with screw loosening detected in follow up imaging. To compare the groups Chi^2^-test was used. *p* = 0.004.

**Table 4:** Bacterial spectrum of cases with screw loosening.

Bacterium	CFR-PEEK group	Matched titanium group
MSSA	3	1
MRSA	1	0
Staph. epi	1	1
Strep. ang	1	0

Bacteria causing initial spondylodiscitis of cases with screw loosening. MSSA = Methicillin sensible *Staphylococcus aureus*; MRSA = Methicillin resistant *Staphylococcus aureus*; Staph. epi. = *Staphyloccous epidermidis*; Strep. angi = *Streptococcus anginosus.*

**Table 5:** Bacteria detected in sonication of explanted CFR-PEEK.

Bacterium Spondylodiscitis	Bacterium Sonication
MSSA	n.p.
MSSA	no bacterium
MSSA	Cut. acnes
MRSA	no bacterium
Staph. epi.	no bacterium
Strep. angi.	n.p.

Bacteria detected in sonication of CFR-PEEK material which was explanted because of screw loosening. In two cases no sonication was performed. In one case because no revision operation was performed. In the other case because surgery had to be stopped before material explantation because of extreme bleeding. Cut. acnes = *Cutibacterium acnes*; MSSA = Methicillin sensible *Staphylococcus aureus*; MRSA = Methicillin resistant *Staphylococcus aureus*; Staph. epi. = *Staphyloccous epidermidis*; Strep. angi. = *Streptococcus anginosus*; n.p. = not performed.

**Table 6:** Bacteria detected in sonication of explanted titanium.

Bacterium Spondylodiscitis	Bacterium Sonication
MSSA	no bacterium
Staph. epi.	no bacterium

After sonication of explanted loosened titanium screws no bacterium was detected.

MSSA = Methicillin sensible *Staphylococcus aureus*; Staph. epi. = *Staphyloccous epidermidis.*

In the CFR-PEEK group in five out of six cases with screw loosening 360° fusion was performed, while in the titanium group this was performed in only one out of the two cases (*p* = 0.464).

### Revision surgery

Operative revision was performed in eight cases of the CFR-PEEK group, but only in two cases of the titanium group (*p* = 0.071; Table 7). Reasons for reoperation in the study group included screw loosening in five cases, of which in two cases a relapse of spondylodiscitis had occurred. Relapse of spondylodiscitis was defined as a combination of recurrent back pain, elevated serum inflammation markers and radiographic signs in MRI- and CT-scans, such as new contrast enhancement of the discs and progressive bony destruction of the endplates. In two cases wound healing disorders and in one case a cerebrospinal fluid (CSF) leak had occurred (Table 7). In both cases of revision surgery due to relapse of spondylodiscitis no bacterium was detected by sonication of the explanted material. Both cases of the matched group were reoperated due to screw loosening (Table 7).

**Table 7:** Reasons for reoperation of CFR-PEEK group and matched titanium group.

	CFR-PEEK group	Matched titanium group	*p* value
Total	8	2	0.071
Screw loosening + relapse disctis	2	0	1.000
Screw loosening − relapse discitis	3	2	0.444
Wound healing disorder	2	0	1.000
CSF leak	1	0	1.000

Number of revision surgery performed in both groups and the reasons for reoperation. Fisher’s exact test was applied. CSF = cerebral spine fluid.

## Discussion

The results from this study of 23 consecutive cases with CFR-PEEK instrumentation for spinal infection suggest that screw loosening occurs more frequently when using CFR-PEEK than titanium for instrumentation of spinal infection.

To the best of our knowledge this is the first study comparing CFR-PEEK instrumentation with titanium in the clinical practice for spondylodiscitis.

### Screw loosening and bacterial adhesion

In Europe, *S. aureus* and coagulase negative *Staphylococcus species* are the most frequent causes of spondylodiscitis^15,16^.

In our study, these bacterial species were also the most frequently detected species in the total cohort of patients as well as in the cohort with screw loosening.

At first sight, in vitro laboratory results might show no statistically significant difference in the adhesion of methicillin sensitive *S. aureus* (MSSA), methicillin resistant *S. aureus* (MRSA) and *S. epidermidis*, on CFR-PEEK compared to titanium^14^. However, if absolute numbers of bacterial adhesion are compared, it becomes clear that bacterial adhesion on titanium is approximately half of the adhesion on CFR-PEEK for all three species (titanium: *MRSA*: 3.3 × 10^6^ colony forming unit (cfu); *S. aureus*: 3.6 × 10^6^ cfu; *S. epidermidis*: 4.0 × 10^6^ cfu; CFR-PEEK: MRSA: 8.7 × 10^6^ cfu; *S. aureus*: 7.6 × 10^6^ cfu; *S. epidermidis*: 6.4 × 10^6^ cfu). The reason that this difference did not reach a statistically significant level may be due to a small number of samples; yet, a statistical trend was shown.

In addition, it was shown in the same work, that the surface of CFR-PEEK has a roughness that is almost twice that of titanium (CFR-PEEK 0.38 ± 0.10 µm, titanium 0.20 ± 0.04 µm). It appears conclusive that bacteria can adhere better to a rougher surface. Hitherto, data from this study may provide a reason why there is more screw loosening after CFR-PEEK instrumentation: bacteria can adhere better to CFR-PEEK and promote the loosening process; an observation that seems to be even stronger in vivo than in vitro.

Not all fiber-reinforced materials share the same characteristics. In the recent years several studies have shown the advantages of bioactive glass-fiber reinforced implants^17^. These implants can be modified in the way to promote ossification^18^ and to inhibit bacterial growth and biofilm formation^19^.

Interestingly, in the CFR-PEEK group there were two cases with relapse of spondylodiscitis, while in the matched titanium group no recurrence was reported. In both cases of relapse spondylodiscitis no bacterium was detected during revision surgery despite sonication. This suggests that there might be another virulence factor aside from bacterial adhesion which in combination with CFR-PEEK promotes infection relapse and screw loosening. The idea that there might be more wear particles generated by CFR-PEEK compared to standard material which could cause tissue inflammation was rejected by studies from the orthopedic field^20,21^.

In only one case the sonication of the explanted material a bacterium could be detected. This bacterium was *Cutibacterium acnes* on CFR-PEEK material from a spondylodiscitis initially caused by MSSA. This implicates that antibiotic treatment successfully eliminated the initial causative bacterium in all cases despite the rough surface.

*Cutibacterium acnes* was identified as one of the main species causing pedicle screw loosening because of subclinical low-grade infection^22–24^.

So low-grade infection of CFR-PEEK may play a role in screw loosening.

### Screw loosening and fusion techniques

In a series of 22 primary spinal tumor cases who underwent spinal fusion using CFR-PEEK only one case of screw loosening was found at 12 months after operation^7^. In our study, for patients with spinal infection screw loosening of CFR-PEEK screws was found more frequently and was registered at an earlier time point (mean 110 ± 81 days) compared to the cases with screw loosening found in the series of tumor cases^7^. Spinal metastases or primary spinal tumors are usually located in the vertebral body, whereas spondylodiscitis affects the intervertebral disc and adjacent endplates. So in tumor surgery the affected vertebrae are not instrumented with pedicle screws^7^, while in our series of spondylodiscitis cases pedicle screws were placed in the infected vertebrae.

Figure 2 shows the CT-scan of a patient, who initially suffered from spondylodiscitis of level L4/5, which was instrumented with CFR-PEEK screws on the levels L4–5 and an intercorporal fusion from lateral (XLIF). After one month instrumentation was extended to S1 and S2 with CFR-PEEK screws because of screw loosening of L5. However, three months afterwards he presented again with screw loosening of L4 and L5 (Fig. 2), what lead to explant of the whole material. Figure 3 shows the CT-scan of a patient who initially suffered from spondylodiscitis of the level T7/8. CFR-PEEK instrumentation involved T5–6–9–10 and a vertebral body replacement of T7/8 from transthoracal. After one month he presented with spondylodiscitis of T10/11. Instrumentation was extended with CFR-PEEK screws to T11–12 and intercorporal fusion was performed from lateral. Six weeks later he presented with extreme screw loosening of T11 and T12 (Fig. 3). Screws of T1 and T12 were removed and instrumentation was extended with titanium screws to L1–2–3. These two cases of severe spondylodiscitis show that screw loosening was especially found where screws were inserted in the affected vertebrae. From our point of view this is a risk factor for screw loosening of CFR-PEEK screws, especially in severe cases of spondylodiscitis. A reason for this may be an ongoing inflammatory reaction leading to a segmental instability.

### Screw loosening and mechanical loading

Inadequate anterior support and osteoporosis are commonly regarded as risk factors for screw loosening after spinal instrumentation^25^. However, number of patients with osteoporosis was even higher in the titanium group than in the CFR-PEEK group. Regarding 360° fusion procedures the groups were equal as it was a matching criterion. Within the cases where screw loosening was observed in the CFR-PEEK group, 83% underwent 360° fusion; suggesting that inadequate anterior support did not play a role in screw loosening after CRF-PEEK instrumentation for spondylodiscitis.

Compared to 108 spondylodiscitis patients with titanium instrumentation operated from January 2014 until December 2016, 360° fusion techniques were used significantly more frequently with CFR-PEEK instrumentation. When compared to titanium CFR-PEEK has a lower elastic module which is similar to that of cortical bone^26–28^. Due to this higher elasticity 360° fusion was used more frequently with CFR-PEEK instrumentation. An elastic module similar to that of cortical bone was considered to be an advantage of CFR-PEEK because it avoids a mechanical mismatch during ossification^2^. In cadaver studies it was shown that CFR-PEEK pedicle screws resisted a similar cyclic loading compared to titanium screws^29^. Successful results of CFR-PEEK cages exist including cases of spinal infection^2^. However, in one third of the reported cases an additional instrumentation with titanium or steel from posterior was performed.

### Strengths

One strength of our study is its novelty and its consecutive series. Follow-up results of a similar cohort of patients treated with CFR-PEEK instrumentation for spondylodiscitis do not exist so far.

### Limitations

One limitation of our study is the fact that it was not a randomized controlled trial. However, with the matched analysis we provided a high level of objective comparability in one center with one treatment standard for spondylodiscitis. Another limitation is the fact that this is a study of a small number of cases. However, even this small number has an impact on clinical practice. It is unlikely that there will be a comparable prospective study with a higher number of cases. Since we know that there is more screw loosening with CFR-PEEK screw in spondylodiscitis cases, this will not be justifiable from an ethical point of view.

## Conclusion

As opposed to other indications like tumor, degeneration and fractures, CFR-PEEK screws show a higher rate of screw loosening than titanium screws in spinal infections in this series with two potentially underlying reasons. A probably stronger bacterial adhesion on CFR-PEEK in vivo as shown by a statistical trend in vitro and instrumentation of spondylytic vertebrae, especially in severe cases of spondylodiscitis.

Until these factors are validated, we advise caution when implanting CFR-PEEK screws in infectious cases; if doing so, instrumentation of spondylitic vertebrae should be avoided.

## Data Availability

Data supporting the findings of this study are available within this article.
